# Interventions by Cardiovascular Drugs Against Aircraft Noise-Induced Cardiovascular Oxidative Stress and Damage

**DOI:** 10.3390/antiox14010059

**Published:** 2025-01-07

**Authors:** Marin Kuntić, Ivana Kuntić, Jiayin Zheng, Leonardo Nardi, Matthias Oelze, Arijan Valar, Dominika Mihaliková, Lea Strohm, Henning Ubbens, Qi Tang, Liyu Zhang, Guilherme Horta, Paul Stamm, Omar Hahad, Dilja Krueger-Burg, Huige Li, Sebastian Steven, Adrian Gericke, Michael J. Schmeisser, Thomas Münzel, Andreas Daiber

**Affiliations:** 1 Laboratory of Molecular Cardiology, Department of Cardiology 1, University Medical Center of the Johannes Gutenberg-University, 55131 Mainz, Germany; marin.kuntic93@gmail.com (M.K.); ivana45@gmail.com (I.K.); zhengjiayin12@outlook.com (J.Z.); matzeoelze@aol.com (M.O.); arijan.valar@gmail.com (A.V.); dominika.mihalik@gmail.com (D.M.); leastrohm94@aol.com (L.S.); henningubbens@gmail.com (H.U.); paul.stamm@unimedizin-mainz.de (P.S.); omar.hahad@unimedizin-mainz.de (O.H.); sebastiansteven@gmx.de (S.S.); tmuenzel@uni-mainz.de (T.M.); 2 German Center for Cardiovascular Research (DZHK), Partner Site Rhine-Main, 55131 Mainz, Germany; huigeli@uni-mainz.de; 3 Institute of Anatomy, University Medical Center of the Johannes Gutenberg-University, 55131 Mainz, Germany; leonardi@uni-mainz.de (L.N.); guihorta@uni-mainz.de (G.H.); dkruegerburg@uni-mainz.de (D.K.-B.); mschmeisser@uni-mainz.de (M.J.S.); 4 Department of Ophthalmology, University Medical Center of the Johannes Gutenberg-University, 55131 Mainz, Germany; qitang.med@gmail.com (Q.T.); liyuzhang0@gmail.com (L.Z.); adrian.gericke@unimedizin-mainz.de (A.G.); 5 Department of Pharmacology, University Medical Center of the Johannes Gutenberg-University, 55131 Mainz, Germany; 6 Department of Cardiology, University Heart Centre Frankfurt, Goethe University Frankfurt, 60590 Frankfurt am Main, Germany

**Keywords:** aircraft noise exposure, high blood pressure, endothelial dysfunction, oxidative stress, inflammation, preventive therapy, beta-blocker, alpha-blocker

## Abstract

Noise pollution is a known health risk factor and evidence for cardiovascular diseases associated with traffic noise is growing. At least 20% of the European Union’s population lives in noise-polluted areas with exposure levels exceeding the recommended limits of the World Health Organization, which is considered unhealthy by the European Environment Agency. This results in the annual loss of 1.6 million healthy life years. Here, we investigated the protective effects of cardiovascular drug interventions against aircraft noise-mediated cardiovascular complications such as elevated oxidative stress or endothelial dysfunction. Using our established mouse exposure model, we applied mean sound pressure levels of 72 dB(A) for 4 d. C57BL/6 mice were treated with the beta-blocker propranolol (15 mg/kg/d s.c. for 5 d) or the alpha-blocker phenoxybenzamine (1.5 mg/kg/d s.c. for 5 d) and noise-exposed for the last 4 d of the drug administration. Short-term noise exposure caused hypertension (measured by tail-cuff blood pressure monitoring) and impaired endothelial function (measured by isometric tension recording in the aorta and video microscopy in cerebral arterioles in response to acetylcholine). Noise also increased markers of oxidative stress and inflammation. Treatment of mice with propranolol and phenoxybenzamine prevented endothelial and microvascular dysfunction, which was supported by a decrease in markers of inflammation and oxidative stress in heart tissue and the brain. Amelioration of noise-induced hypertension (systolic blood pressure) was not observed, whereas pulse pressure was lowered by trend. This study provides a novel perspective mitigating the adverse effects of noise pollution, especially in vulnerable groups with medication, a rationale for further pharmacological human studies.

## 1. Introduction

With recent medical care and hygiene advances, non-communicable diseases (NCDs) have taken the forefront of the total disease burden worldwide [1]. With this increase in NCDs came a whole new class of environmental risk factors, one of which is traffic noise. The importance of traffic noise as a risk factor arose from the recent urban expansion and increase in population density. In the European Union, more than 20% of the population is estimated to live in areas where the transportation noise exceeds 55 dB (L_den_ = average sound pressure level over the whole day—day, evening, night—with a penalty 5 dB(A) for evening noise and 10 dB(A) for nighttime noise) [2]. Aircraft noise is especially detrimental, as the intermittence and randomness of the high dB sound pressure cause annoyance and sleep disturbance [2,3]. Traffic noise, in general, was shown to be associated with an increased risk of cardiovascular diseases, such as ischemic heart disease [2], heart failure [4], stroke [5], hypertension [6], and overall cardiovascular mortality [7]. Nighttime aircraft noise was previously shown to impair endothelial function and increase blood pressure in patients with coronary artery disease [8]. This provides evidence for the detrimental effect of noise in patients with preexisting cardiovascular disease.

The mechanism by which noise influences the cardiovascular system starts as a stress response, activating the hypothalamic–pituitary–adrenal (HPA) axis and the sympathetic nervous system (SNS) [9,10]. Activation of the SNS results in the release of catecholamines, such as adrenaline and noradrenaline, which can cause vasoconstriction, inflammation, and vascular oxidative stress [11]. Activation of the SNS and the release of catecholamines were previously observed in noise-exposed mice and humans [12,13]. Chronic increase in catecholamine release can lead to arterial hypertension, myocardial dysfunction, and ventricular remodeling [14]. Alpha (α)- and/or beta (β)-blockers are commonly used cardiovascular pharmacological interventions for the treatment of hypertension, congestive heart failure, acute myocardial infarction, arrhythmias, and other heart insufficiencies [15,16]. These pharmacological interventions block α- and β-adrenergic receptors to which catecholamines bind, decreasing vascular resistance and blood pressure, thereby restoring heart function [17]. There is a general lack of data on the potential positive effects of α- and β-blockers on noise-induced cardiovascular side effects. One study has established that alpha-blockers attenuate anxiety levels and restore impaired spatial memory of noise-exposed rats [18], which was attributed in part to improved blood pressure. Animal data also revealed that chronic noise exposure causes a sustained increase in blood pressure in monkeys (industrial noise with L_eq_ 85 dB(A) (L_eq_ = the total sound energy determined over the course of a measurement) and peak sound levels 97 dB(A) for 9 months) [19] or impairment of vascular function in rats (100 dB(A) synthetic noise for various duration) [20,21].

The above-described mode of action of α- and β-blockers, as well as reports on a significant association between aircraft noise levels at night and antihypertensive medication in the UK (OR = 1.43, 95% confidence interval (CI) 1.19–1.73 for a 10 dB(A) increase in L_night_ = average sound pressure level during the night), refs. [22,23] prompted us to investigate the suitability of these drugs for mitigation of adverse noise health effects. Also, the known antioxidant effects of α- and β-blockers [24,25] provide an attractive basis for the use of these drugs in aircraft noise-exposed mice. With the present study, we wanted to explore the possible improvements to vascular dysfunction caused by aircraft noise exposure. We also aimed to elucidate the connection between the brain and the cardiovascular system, with oxidative stress and inflammation as the main mediators.

## 2. Materials and Methods

### 2.1. Animal Handling and Exposure

C57BL/6 mice, aged 8–12 weeks, of male sex, were used in the experiments. The mice were obtained from Charles River (Sulzfeld, Germany) and housed for 2–3 weeks prior to the experiment under a 12 h light/dark cycle in the animal facility of the University Medical Center Mainz, and were fed standard rodent chow (#1126, Ssniff, Soest, Germany) ad libitum. All treatments were performed in accordance with the Guide for the Care and Use of Laboratory Animals as adopted by the U.S. National Institutes of Health, and approval was granted by the Ethics Committee of the University Medical Center Mainz and the Landesuntersuchungsamt Rheinland-Pfalz (Koblenz, Germany; permit number: 23 177-07/G 18-1-084). The aircraft noise protocol was used as described before [13], here detailed in brief: The aircraft noise consisted of a two-hour-long recording of airplane takeoff and landing events that were randomly spaced to prevent adaptation. The maximum sound pressure level was 85 dB(A), and the mean sound pressure level was 72 dB(A). The sound file was provided by the German Ministry of Transport and was optimized by a sound engineer (Schalltechnisches Ingenieurbüro Pies GbR, Boppard, Germany). The aircraft noise exposure was performed 24 h per day for a total of 4 days to mimic acute noise effects that previously showed significant functional and biochemical changes in mice, whereas exposure to white noise at the same sound pressure level caused none of these changes [13,26]. In addition, the same noise protocol caused changes in endothelial function and stress hormone levels in humans after one night of exposure [8,27]. The mean sound pressure level of 72 dB(A) was selected since it roughly represents the noise generated by a passenger car or a train passing by [10]. It is loud enough to ensure functional and biochemical changes within a short time window but is far below the majority of previous animal studies using peak sound pressure levels higher than 100 dB(A) or mean sound pressure levels of 85 dB(A) and higher, which will ultimately cause inner ear damage and hearing loss besides the indirect noise effects we were interested in [10,28].

**Figure 1:**
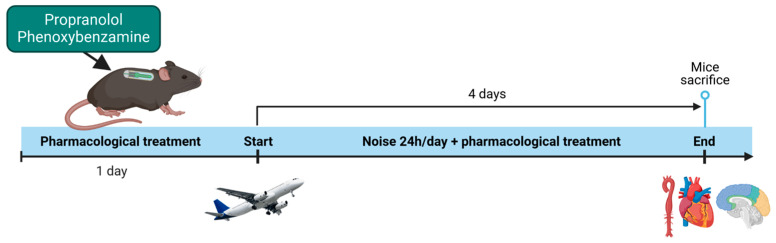
Schematic representation of the exposure and treatment paradigm.

For the pharmacological treatment, mice were administered with either propranolol (Merck, Darmstadt, Germany) 15 mg/kg/d and thus in a concentration sufficient to markedly reduce heart rate as shown previously [29,30], or phenoxybenzamine (Sigma-Aldrich, Darmstadt, Germany) 1.5 mg/kg/d. Propranolol was chosen as an unselective β-blocker to also ensure inhibition of β_2_- and β_3_-receptor-mediated actions that may play a dominant role for immune cell activation (see Section 4.3). Phenoxybenzamine was chosen as it effectively prevents α-receptor agonist-dependent vasoconstriction [31] and is a long-acting α-receptor antagonist that creates a “chemical sympathectomy”, ensuring complete α-receptor blockade. Both drugs were administered via osmotic minipumps (Alzet, Cupertino, CA, USA, 1007D, 0.5 μL per hour) and implanted subcutaneously as described previously [32]. An incision of approximately 1 cm was made on the dorsal flank, and the mini osmotic pump was inserted. The incisions were sutured and were treated with lidocaine ointment for pain relief. Mice were allowed 1 day to rest before being exposed to aircraft noise. After the last day of noise exposure, mice were sacrificed under deep ketamine/xylazine anesthesia and tissues were harvested. A summary of the treatment protocol is depicted in Figure 1.

### 2.2. Non-Invasive Blood Pressure Measurement

Non-invasive tail-cuff plethysmography (CODA—Kent Scientific, Torrington, CT, USA) was used to measure the mice’s blood pressure as described previously [26]. Briefly, the mice were restrained in plastic restraining tubes and warmed up on a heating plate while covered to induce darkness. Two cuffs were placed on the tail of the mouse: one occlusion cuff and one volume pressure-recording cuff. Ten measurements were performed on each mouse, and the mean value was used. Mice were trained in the blood pressure machine at least two times before the official measurements were obtained.

### 2.3. Vascular Function by Isometric Tension Measurement and Video Microscopy

The procedure for the isometric tension study of the isolated aortic rings was previously described in detail [13,26]. Briefly, a 4 mm segment of the thoracic aorta was excised and cleaned of any perivascular adipose tissue. The cleaned aortic pieces were then suspended in the organ bath chamber from force transducers to measure the force exerted by the smooth muscle. Organ bath chambers had a constant flow of carbogen gas (95% oxygen and 5% carbon dioxide *v*/*v*) and were kept at 37 °C. Cyclooxygenase inhibitor indomethacin was added to the chambers to prevent prostaglandin and eicosanoid production that might interfere with the relaxation measurements. Aortas were reconstructed with prostaglandin F2α (yielding approximately 80% of the maximal tone induced by KCl bolus) and then subjected to titration of either acetylcholine (ACh) and nitroglycerin (NTG) to induce endothelium-dependent and -independent relaxation, respectively.

Cerebral arteriole isolation has recently been detailed in our previous studies [33,34]. In summary, following the isolation of the brain, we meticulously isolated the vascular tree of the middle cerebral artery (MCA) using fine-point tweezers and microscissors. The MCA and its branches were then transferred to a pressure myograph. The main branch of the MCA was cannulated with a micropipette, guiding the pipette tip into a branching arteriole by gently pulling the cannulated end of the MCA while pushing the free end against the pipette tip. After successful cannulation, the free end of the MCA was secured to the micropipette. The non-cannulated end of the arteriole was then tied to a second micropipette, and the pipette tips were separated to gently stretch the arteriole. Once positioned on an inverted microscope, the arteriole was pressurized to 40 mmHg through the indwelling micropipette and visualized using a digital camera. The organ chamber maintained a constant temperature of 37 °C and a flow of carbogen gas (95% oxygen, 5% CO_2_ *v*/*v*) throughout the experiment. Arteriole viability was evaluated by adding KCl (100 mM) to the circulating bath solution. Following a 45 min equilibration period, we applied the thromboxane mimetic U46619 (10^−11^ to 10^−6^ M; Cayman Chemical, Ann Arbor, MI, USA) cumulatively to the circulating bath. Upon completion of the concentration–response curve, U46619 was washed out, and the arteriole was preconstricted to 50–70% of its initial diameter through titration of U46619. Subsequently, we measured the responses to the endothelium-dependent vasodilator acetylcholine and the NO donor sodium nitroprusside (both 10^−9^ to 10^−4^ M; Sigma-Aldrich, Munich, Germany) via cumulative application into the organ chamber.

### 2.4. Dihydroethidium Fluorescent Microtopography

The method was previously used in our lab and described in detail [13,35]. Briefly, a 4 mm piece of thoracic aorta or brain tissue including a cerebral arteriole was excised and embedded in optimal cutting temperature (OCT) resin (TissueTek^TM^, Sakura Finetek, Umkirch, Germany) and frozen in liquid nitrogen. Frozen blocks were cut on a cryo-microtome to a thickness of 8 µm and placed on microscopy slides (SuperFrost^®^—VWR International, Darmstadt, Germany). Slides were then incubated with 1 µM dihydroethidium (DHE) for 30 min at 37 °C. After the incubation, slides were washed with cold PBS and imaged under a fluorescent microscope (Axiovert 40CFL with Axiocam MRm, Zeiss, Jena, Germany) with the excitation wavelength of 510–520 nm and emission wavelength of 580–610 nm. The fluorescence images were quantified by pixel intensity using ImageJ software version 1.54k.

### 2.5. Western Blot

Protein expression in selected tissues was measured according to the standard western blot procedure as described [13]. Briefly, proteins were isolated from heart and brain tissue and protein concentration was determined using the Bradford assay. Proteins were loaded onto a polyacrylamide gel and voltage was applied to move the proteins in the electric field. After separation, proteins were blotted onto a nitrocellulose membrane by applying current and stained by Ponceau-S dye to identify cutting patterns. Primary antibodies were used to identify the following proteins of interest: VCAM-1 (1:200, Santa Cruz #sc-13160, Dalas, TX, USA), NOX2 (gp91phox, 1:500, BD Bioscience #611415, Franklin Lakes, NJ, USA) HO-1 (1:2000, abcam #ab1220-50, Cambridge, UK), P-MARCKS^Ser152/156^ (1:1000, Cell Signaling #2741, Danvers, MA, USA), IL-6 (1:1000, abcam #ab208113, Cambridge, UK), P-p47phox^Ser328^ (NCF-1 (Phospho-Ser328), 1:500, Assay Biotech #A1161, Sunnyvale, CA, USA), CD68 (1:1000, abcam #ab31630, Cambridge, UK), and nNOS (1:500, BD Bioscience #610309, Franklin Lakes, NJ, USA).

Alpha (α)-actinin or beta (β)-actin (1:2500 each, Sigma-Aldrich #A5044 and #A5060, St. Louis, MO, USA) were used for assessing total protein loading and transfer. Secondary anti-mouse or anti-rabbit antibody was conjugated to the horseradish peroxidase (1:10,000 each, Vector Lab. #PI-2000 (anti-mouse IgG) and #PI-1000 (anti-rabbit IgG), Burlingame, CA, USA). Protein expression was quantified densitometrically with a chemiluminescence imager (Chemostar Imager—Intas Science Imaging Instruments GmbH, Göttingen, Germany) by using the Gel-Pro Analyzer software version 6.3.

### 2.6. Dot Blot

Expression of the circulating inflammation marker MCP-1 was quantified in mouse plasma by a dot blot technique [13]. Briefly, mouse plasma (2 µL) was diluted 1:100 in PBS and applied directly to a nitrocellulose membrane using a Minifold 1 vacuum dot blot system (Whatman^®^ Schleicher & Schuell GmbH, Hamburg, Germany). The membrane was washed twice, removed from the dot blot system, and stained with Ponceau-S dye for loading control. A primary antibody for MCP-1 (1:10,000, Biorad #AAM43, Feldkirchen, Germany) was used together with an anti-rabbit secondary antibody (1:10,000, Vektor Lab. #PI-1000, Burlingame, CA, USA). Protein expression was quantified densitometrically with a chemiluminescence imager (Chemostar Imager—Intas Science Imaging Instruments GmbH, Germany) and using the Gel-Pro Analyzer software version 6.3.

### 2.7. Statistical Analysis

All statistical analyses were performed in Prism software for Windows, version 9 (GraphPad Software LLC, La Jolla, CA, USA). *p*-values were obtained using one-way ANOVA for all bar graphs and two-way ANOVA for the relaxation curve graphs, with Tukey’s post-hoc comparison of multiple means. *p*-values lower than 0.05 were used to indicate statistical significance. All data are presented as mean ± standard deviation (SD) or standard error of the mean (SEM) as indicated in the figure legends. The number of replicates in the different assays may vary since not all animals were used in all assays.

## 3. Results

### 3.1. Effects of Pharmacological Interventions on Noise-Induced Hypertension and Endothelial Dysfunction

**Figure 2:**
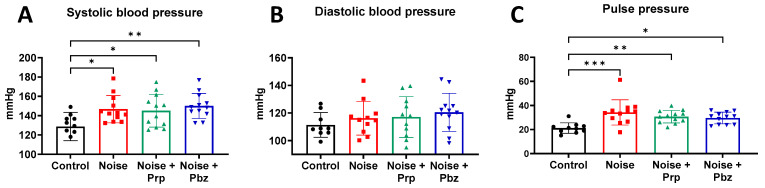
Effects of pharmacological interventions on noise-induced hypertension. (**A**) Systolic, (**B**) diastolic, and (**C**) pulse blood pressure on the final day of noise exposure. The results are presented as jitter plots with mean ± SD, where dots show the number of independent measurements per group. Significance is indicated as * and ** and *** when *p* < 0.05 and *p* < 0.01 and *p* < 0.001, respectively. Prp = propranolol; Pbz = phenoxybenzamine.

Aircraft noise exposure increased systolic blood pressure and it remained elevated even after the treatment with propranolol or phenoxybenzamine (Figure 2A). Even though diastolic blood pressure did not significantly change, it was increased by trend with noise exposure, and no improvement was observed after pharmacological intervention (Figure 2B). Pulse pressure was the only blood pressure parameter that showed a significant increase after noise, which was improved by trend with both drug treatments (Figure 2C).

**Figure 3:**
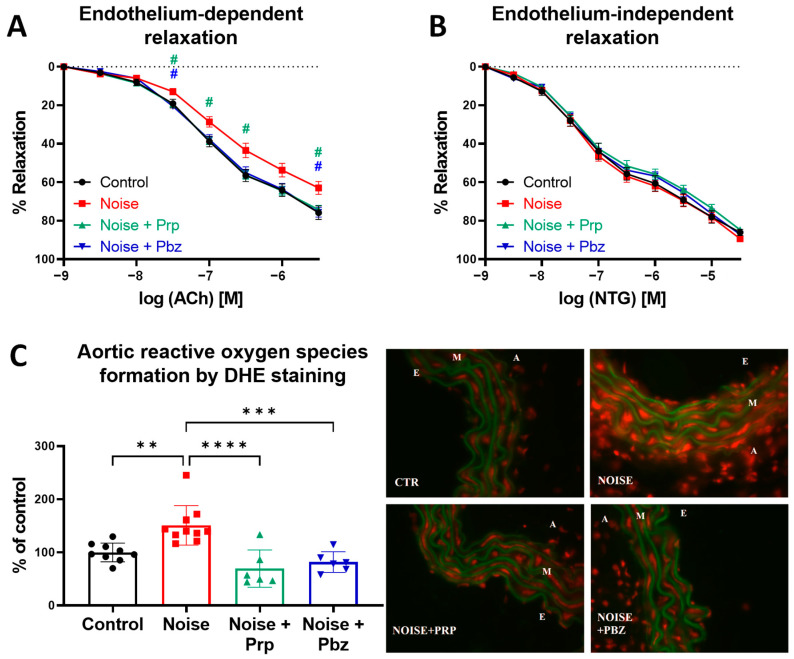
Effects of pharmacological interventions on noise-induced ROS formation and endothelial dysfunction in the aorta. (**A**) Endothelium-dependent relaxation in the presence of acetylcholine (ACh) and (**B**) endothelium-independent relaxation in the presence of nitroglycerin (NTG) of thoracic aortic rings, as measured by isometric tension method. The results are presented as mean ± SEM of *n* = 10–16 aortic rings per group. Significance is indicated as # when *p* < 0.05 between the noise-exposed group and pharmacologically treated groups. (**C**) Staining of aortic cryosections with dihydroethidium with representative images that show ROS formation (red fluorescence) and aortic lamina (green autofluorescence). A—adventitia, M—media, E—endothelium. The results are presented as jitter plots with mean ± SD of *n* = 6–10. Significance is indicated as ** and *** and **** when *p* < 0.01 and *p* < 0.001 and *p* < 0.0001, respectively. Prp = propranolol; Pbz = phenoxybenzamine.

The acetylcholine (ACh)-dependent relaxation curve of thoracic aortic rings was shifted to the right after noise exposure, indicating endothelial dysfunction, which was mitigated by both pharmacological interventions (Figure 3A). No change was observed in the nitroglycerin (NTG)-dependent relaxation curves, indicating no effect on the smooth muscle cells and endothelium-independent relaxation (Figure 3B). Oxidative stress is one of the most common reasons for impairment of vascular signaling. Noise was previously shown to increase vascular oxidative stress in mouse models [13,26]. Also in the present study, aortic reactive oxygen species formation was increased by noise exposure. Both drugs, propranolol and phenoxybenzamine, mitigated the noise-induced total ROS production in aortic cryosections, as envisaged by DHE staining (Figure 3C). The ROS production after treatment with propranolol even reached a level below the control group by trend.

### 3.2. Effects of Pharmacological Interventions on Noise-Induced Microvascular Dysfunction

**Figure 4:**
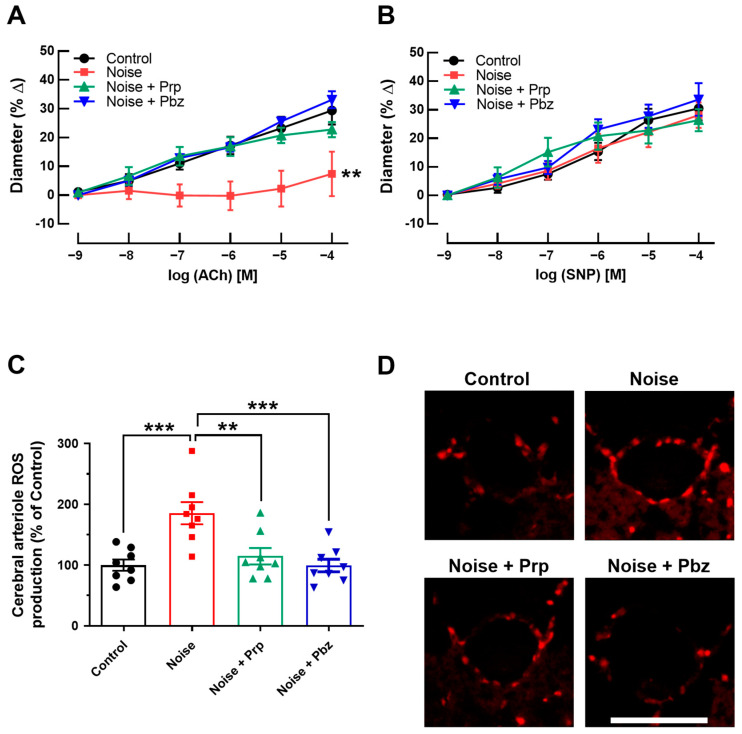
Responses of cerebral arterioles to vasodilators. (**A**) Responses to the endothelium-dependent vasodilator acetylcholine were impaired in arterioles from mice exposed to noise, indicative of endothelial dysfunction. In contrast, the β-blocker propranolol and the α-blocker phenoxybenzamine prevented vessels from noise-induced endothelial dysfunction. (**B**) Neither noise exposure nor additional treatment by propranolol or phenoxybenzamine affected endothelium-independent vasodilation induced by sodium nitroprusside. (**C**) A marked increase in ROS levels (as indicated by DHE staining intensity) was observed in the vascular wall of cerebral arterioles from noise-exposed mice. Remarkably, propranolol and phenoxybenzamine prevented ROS formation. (**D**) Representative pictures of DHE-stained cerebral arterioles’ cross-sections. The results are presented as mean ± SEM of *n* = 8 arterioles per group (** *p* < 0.01; *** *p* < 0.001). Scale bar = 20 µm. Prp = propranolol; Pbz = phenoxybenzamine; SNP = sodium nitroprusside.

ACh-dependent dilation was markedly impaired in cerebral arterioles of noise-exposed mice, indicative of endothelial dysfunction. Remarkably, both propranolol and phenoxybenzamine restored endothelial function following noise exposure (Figure 4A). In contrast, vasodilation in response to the endothelium-independent vasodilator sodium nitroprusside (SNP) was similar in both groups, suggesting that smooth muscle function was not affected by noise exposure or pharmacological treatment (Figure 4B). Noise exposure induced ROS formation in cerebral arterioles as reflected by elevated DHE staining intensity (Figure 4C,D). In contrast, treatment with propranolol and phenoxybenzamine blocked ROS formation (Figure 4C,D).

### 3.3. Effects of Pharmacological Interventions on Protein Markers of Noise-Induced Oxidative Stress and Inflammation

**Figure 5:**
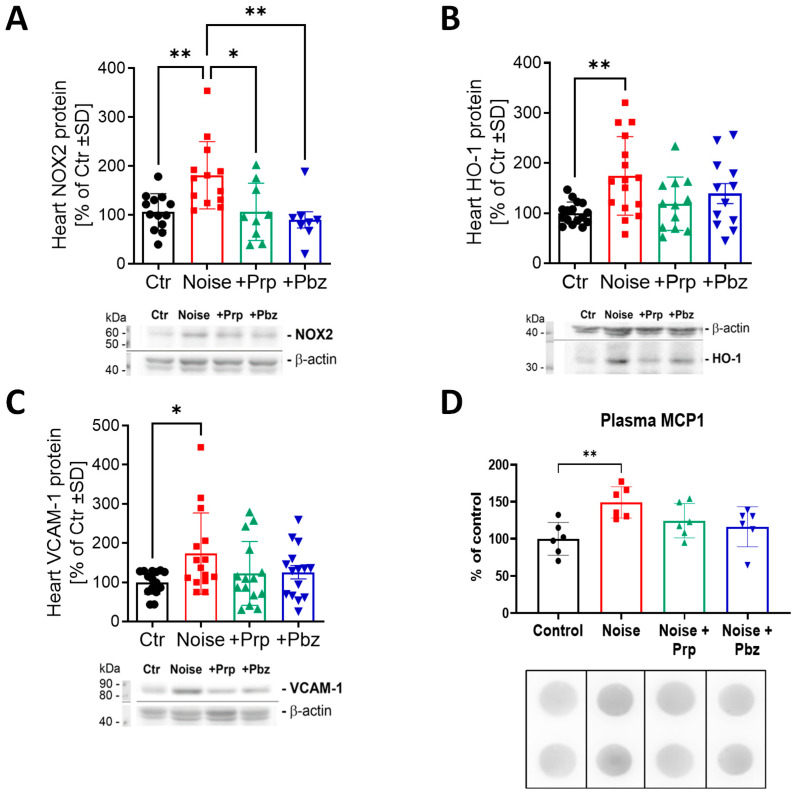
Protein markers of oxidative stress and inflammation in heart tissue and plasma. Protein expression of (**A**) NOX2, (**B**) HO-1, and (**C**) VCAM1 in cardiac tissue was determined by western blot analysis. (**D**) Protein expression of MCP-1 in plasma was determined by dot blot analysis. Representative images are shown below the respective densitometric quantification. The results are presented as jitter plots with mean ± SD of *n* = 6–14. Significance is indicated as * and ** when *p* < 0.05 and *p* < 0.01, respectively. Prp = propranolol; Pbz = phenoxybenzamine.

The increase in protein expression of NOX2 in cardiac tissue after noise exposure was significantly reduced after treatment with both propranolol and phenoxybenzamine, indicating protection against oxidative stress (Figure 5A). HO-1, an important antioxidant enzyme and a marker of nuclear factor erythroid 2-related factor 2 (NRF2) expression, was significantly increased by noise, indicating stress-induced antioxidant defense upregulation, which was again partially normalized by propranolol and phenoxybenzamine (Figure 5B). Both pharmacological interventions also prevented significant upregulation of the inflammation marker VCAM1, which was significantly increased in the noise-exposed group (Figure 5C). A similar pattern was observed in the circulating levels of MCP-1, showing a possible presence of systemic inflammation after noise exposure and successful mitigation by both pharmacological treatments (Figure 5D).

**Figure 6:**
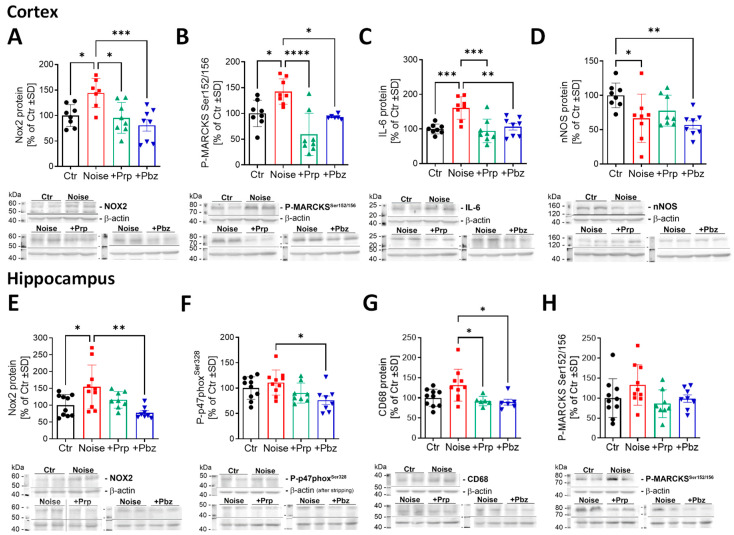
Protein markers of oxidative stress and inflammation in brain tissue. Protein expression of (**A**) NOX2, (**B**) P-MARCKS^Ser152/156^, (**C**) IL-6, and (**D**) nNOS in the cortex and (**E**) NOX2, (**F**) P-p47phox^Ser328^, (**G**) CD68, and (**H**) P-MARCKS in the hippocampus were determined by western blot analysis. Representative images are shown below to the respective densitometric quantification. The results are presented as jitter plots with mean ± SD of *n* = 7–10 for each brain region. Significance is indicated as * and ** and *** and **** when *p* < 0.05 and *p* < 0.01 and *p* < 0.001 and *p* < 0.0001, respectively. Ctr = control; Prp = propranolol; Pbz = phenoxybenzamine.

Protein expression in brain tissues showed an increase in oxidative stress and inflammation markers NOX2, P-MARCKS, and IL-6 as well as a decrease in nNOS involved in cognitive function in the cortex (Figure 6A–D). The oxidative stress markers NOX2 (catalytic subunit of the phagocytic NADPH oxidase), P-p47phox (regulatory cytosolic subunit of NOX2), and P-MARCKS (marker of protein kinase C activation) were also upregulated, at least by trend, by noise in the hippocampus and the marker of inflammation CD68 showed a similar trend of increased expression (Figure 6E–H). Whereas all of these markers were significantly altered by noise exposure in the cortex, α- or β-blocker interventions prevented a significant change in this brain region, and for some targets (NOX2, P-MARCKS, and IL-6) showed a significant reduction for both drugs. Although the overall effects, by noise and the drugs, were somewhat less pronounced in the hippocampus, also here at least one of the drugs caused significant reduction in the expression of the different targets (NOX2, P-p47phox, and CD68). Only the decreased nNOS expression in the noise-exposed mice was not affected by the therapy.

## 4. Discussion

**Figure 7:**
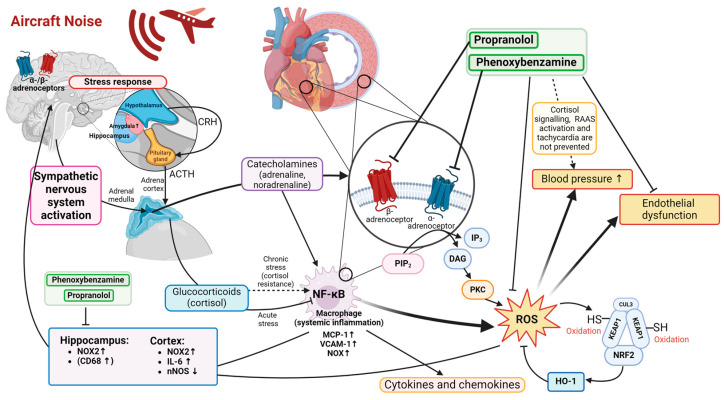
Mechanistic overview scheme of the effects of aircraft noise on endothelial function. Noise acts on the stress response pathway, activating the sympathetic nervous system and the hypothalamic–pituitary–adrenal (HPA) axis through the activation of the amygdala and the hypothalamus, by releasing corticotropin-releasing hormone (CRH) into the pituitary gland, further stimulating the release of adrenocorticotropic hormone (ACTH) into the circulation [36]. Sympathetic nervous system activation induces inflammation and oxidative stress through catecholamine release. Catecholamines bind to α- and β-adrenergic receptors in the brain, on immune cells, and on vascular/cardiac tissue, which promote downstream activation of ROS-releasing enzymes and mediators of inflammation, leading to endothelial dysfunction. Treatment with α- or β-receptor blockers reduced markers of inflammation and oxidative stress in both the cardiovascular and brain tissue and alleviated the negative effects on the endothelial function. Some of the depicted pathways were summarized from previous work [10]. Arrows up or down nearby protein names mean up- or downregulation of protein. Created with BioRender.com.

In the present study, we explored the suitability of α- and β-blocker pharmacological interventions to mitigate the adverse effects of aircraft noise exposure. We found that the pharmacological intervention with propranolol or phenoxybenzamine could mitigate the noise-induced endothelial dysfunction in both the aorta and cerebral arterioles. However, no improvement in blood pressure was observed. Both pharmacological interventions reduced vascular oxidative stress in both the aorta and cerebral arterioles, as shown by DHE staining, and also normalized protein markers of oxidative stress in cardiac and cerebral tissues. Systemic, cardiac, and cerebral inflammation was also prevented by both treatments, showing potent anti-inflammatory effects. The major findings are summarized in Figure 7.

### 4.1. Effects of Noise on Oxidative Stress and Inflammation

Noise, as a risk factor, is interconnected to cardiovascular diseases, such as ischemic heart disease, heart failure, stroke, and hypertension [7,10,37]. Most cardiovascular diseases share molecular mechanisms that involve oxidative stress and inflammation, which are intricately linked and drive each other in a vicious circle [38]. Oxidative stress has many implications for the cardiovascular system, as it can disrupt vascular nitric oxide signaling through the uncoupling of endothelial nitric oxide synthase and the scavenging of nitric oxide by superoxide radicals [39]. The role of oxidative stress in aircraft noise-induced vascular dysfunction was shown in an interventional study where the noise-induced reduction in flow-mediated dilation, a surrogate measurement of endothelial function, was recovered by vitamin C treatment [27]. Importantly, human studies have reported elevated levels of circulating catecholamines associated with increased traffic noise exposure [40,41]. It was previously shown in human subjects that noise causes vascular inflammation by activating the amygdala and the limbic system leading to more cardiovascular events [42]. A study on nighttime train noise showed that the plasma proteome of healthy individuals displayed activation of pro-inflammatory pathways [43].

### 4.2. Effects of α- and β-Blockers on the Stress Response

Previous work showed that phenoxybenzamine improved restraint stress-induced mental stress conditions [44] and beneficially influenced T cell suppression and enhancement of B cell function [45]. Propranolol may decrease fear expression by altering network-correlated activity and by weakening the reactivation of the initial traumatic memory trace [46]. Propranolol also prevented restraint stress-dependent tumor growth [47]. Since noise produces a stress response similar to other types of mental stress, it would be plausible that the treatment with α- and β-blockers would show protective effects in cases of mental stress. A study conducted on 30 patients in which subjects were shown disturbing images to induce mental stress showed that the administration of propranolol before the stress resulted in lower systolic blood pressure and lower salivary alpha-amylase, a marker of sympathoadrenal medullar activation, compared to the placebo-treated subjects [48]. Another study on acute mental stress showed improvement of heart rate and circulating IL-6 levels in the propranolol-treated group in comparison to placebo, indicating the role of SNS activation in the inflammatory response to stress [49]. Interestingly, the effect on inflammation was only observed in men, not women. Other studies have also observed improvements in stress-induced immune modulation by β-blockers [50]. An interesting interventional study showed that the administration of β-blockers, but not α-blockers, rescued the mental stress-induced impairment of the vascular function index (ratio of relaxation by sodium nitroprusside and metacholine) [51]. Improvement in mental stress-induced vascular function was observed in another study, where the administration of propranolol and metoprolol both alleviated changes in systemic vascular resistance [52]. An early survey of 15 hypertensive patients showed that no significant improvement in blood pressure was observed in the β-blocker treatment group after the mental stress task. However, a reduction in heart rate was observed in one of the administered mental stress tests [53]. In addition, another study showed no impact of β-blocker treatment on circulating catecholamine levels after either mental or physical stress [54]. Taken together, these studies show that adrenergic blockade, especially by β-blockers, can alleviate some of the adverse cardiovascular and inflammatory effects of acute mental stress, but the relationship is not yet clear. Furthermore, β-blockers have been shown to have certain positive neuropsychiatric effects on migraines, tremors, anxiety and posttraumatic stress syndrome, aggression, and obsessive-compulsive disorder [55].

### 4.3. Mechanisms of Noise-Induced Oxidative Stress and Inflammation in the Cardiovascular System

We have shown many times in our previous work on animal models that aircraft noise induces oxidative stress and inflammation and impairs vascular function [13,26,56,57]. We have also demonstrated that the adverse effects of aircraft noise on the cardiovascular system start from the brain, where the stress response activates the HPA axis and the SNS [36,58]. Activated SNS leads to an increased release of catecholamines, most notably adrenaline and noradrenaline, which promote inflammation and oxidative stress, e.g., by pro-inflammatory activity of the macrophage β1-receptor [10,59]. Catecholamines may directly modulate the release of pro- or anti-inflammatory cytokines, e.g., suppression by IL-17 via the β2-receptor, by macrophages and other immune cells via interactions with the α- and β-adrenergic receptors [60,61]. Immobilization stress-induced NF-kB activation in monocytes is mediated by α1- and β-adrenoceptors [62]. Noradrenaline is also known to directly stimulate the release of pro-inflammatory cytokines by vascular endothelial cells, an effect mediated by β2-receptors [63]. Alpha1-blockers may reduce inflammation by lowering chemokine production [64]. Activated macrophages lead to the onset and development of atherosclerotic plaques, where they produce oxidative stress through ROS production by phagocytic NADPH oxidase and mitochondria produce electrophilic stress through oxidation of catecholamines by monoamine oxidases, generating toxic aldehydes as well as hydrogen peroxide [65,66]. Therefore, our observations on the beneficial effects of α- and β-blockers against adverse noise effects on the cardiovascular system align with the expectations. Both the reduction in oxidative stress and inflammation are in agreement with the observed improvement in vascular function, as the inhibition of catecholamine binding to the adrenergic receptors prevents their vasomodulatory effects [67]. Importantly, mental stress causes oxidative stress in the brain [68], and pro-inflammatory monocytes can travel to the brain causing ‘‘sickness behavior”, resulting in a vicious cycle. As discussed in our previous review article [69], social stress leads to the release of pro-inflammatory monocytes from the bone marrow and this effect is mediated by β3-receptors. As propranolol is an unselective beta-blocker, it may prevent the effects of all three beta-receptors mentioned above and thereby significantly attenuate noise-induced inflammatory conditions.

The puzzling finding of the present study is that the administration of either propranolol or phenoxybenzamine did not improve the noise-induced increase in blood pressure. It was expected that medication commonly used to treat hypertension would alleviate the blood pressure increase after noise exposure, especially since the mechanisms of catecholamine signaling restriction would be expected to help in sympathetically driven hypertension. However, previous observations in humans subjected to an auditory stimulus showed that atenolol was ineffective in preventing the noise-induced rise in blood pressure [70]. One of the possible explanations is the initiation of reflex tachycardia in the α-blocker treatment, as lowered blood pressure activates the baroreceptor reflex, increasing catecholamine secretion that then acts on the β-adrenergic receptors of the heart, leading to increased heart rate [71]. One of the limitations of this study is that the heart rate of mice was not measured. Thus, we are unable to confirm the existence of tachycardia. Also, the plasma concentrations of the drugs were not determined to confirm successful delivery, which represents a clear weakness due to limited access to LC-MS methodology for these measurements. The start of drug therapy one day before the noise exposure could be somewhat too short to prevent the negative effects.

On the other hand, propranolol reduces blood pressure mainly through chronotropic and inotropic inhibitory mechanisms, and lower cardiac output is often compensated by peripheral vasoconstriction to maintain blood pressure [72]. This conjecture is also subject to limitations due to the need for heart rate data. The chosen propranolol concentration, however, has been repeatedly demonstrated to markedly reduce the heart rate in previous experimental studies [29,30].

Another possible explanation is that either the α- or β-blockade fails to prevent downstream activation of the RAAS, a significant driver of the development of hypertension [73], potentially because catecholamines can directly activate the RAAS [25]. Moreover, catecholamines by inducing oxidative stress via monoamine oxidases could cause blood pressure increases independent of the α- and β-blockade, e.g., as shown by decreased blood pressure in MAO-A/B-deficient mice also displaying other abnormal hemodynamic responses to vasoconstrictors phenylephrine or angiotensin-II [74]. Additionally, inhibition of catecholamine signaling alone, which is only one component of the stress response, is not enough since cortisol/corticosterone signaling is still unaltered. Cortisol was shown to cause endothelial dysfunction under mental stress conditions [75] and to suppress the production of vasodilators such as prostacyclin and nitric oxide [76], all of which could negatively affect blood pressure. Therefore, the improvement in vascular function can be attributed to the reduction in oxidative stress and inflammation, as these are the main drivers of impaired endothelial signaling.

Importantly, pulse pressure (also known as pulse amplitude), calculated as the difference between the systolic and diastolic blood pressure, was decreased by trend by both drug treatments. The systemic pulse pressure is approximately proportional to stroke volume and represents an accepted parameter to indicate the elasticity of vessels, the burden of plaque material diminishing the vessel diameter [77]. Accordingly, lower pulse pressure indicates better vascular function and reduced arterial stiffness [78].

### 4.4. Mechanisms of Noise-Induced Oxidative Stress and Inflammation in the Brain

Several common markers for oxidative stress such as the phagocytic NADPH oxidase catalytic subunit NOX2 (previously termed gp91phox) and its cytosolic regulatory subunit p47phox (in the activated phosphorylated state), and for inflammation such as CD68 and IL-6, were upregulated by noise exposure at the protein level, at least by trend. The known activating pathway for the phagocytic NADPH oxidase via protein kinase C (PKC) was activated by noise as envisaged by the PKC phosphorylation target phospho-MARCKS. In line with a neuronal activation due to the noise-dependent stress response, the neuronal nitric oxide synthase (nNOS) was downregulated in the cortex as previously reported by us [26,79], partially explaining the impairment of cognitive function in highly noise-exposed school children [80,81]. All of these adverse effects of noise on the brain were normalized, at least by trend, by therapy with α- and β-blockers, except the impaired nNOS expression. This may also be of relevance for prevention of dementia, especially Alzheimer’s disease, since the risk for neurodegenerative diseases is exacerbated with increasing traffic noise exposure levels [82,83]. Noise-induced dementia was also mechanistically explained via induction of neuroinflammation and amyloid-beta pathology in noise-exposed Alzheimer’s disease-prone mice [84].

Noise also impaired microvascular function of cerebral arterioles and increased oxidative stress in these microvessels. Exacerbated formation of reactive oxygen species and microvascular dysfunction were improved by both therapies, α- and β-blockers. This is in line with previous mitigation measures applied to noise-exposed mice such as physical exercise and intermittent fasting, both leading to an AMP-activated protein kinase-mediated suppression of oxidative stress and inflammation, being highly effective in also preventing microvascular dysfunction in the brain in response to noise [85]. The improvement in microvascular dysfunction by α- and β-blockers in noise-exposed mice is an important clinical finding, since we have previously shown that noise-inflicted damage is more persistent in micro- than in conductance vessels [57]. Noise cessation for a few days resulted in a recovery of endothelial function in conductance vessels within a few days, which was however not observed for cerebral arterioles. Accordingly, our findings may have implications for patients at risk for stroke who are under standard therapy with α- and β-blockers since previous association studies have shown a higher risk of stroke with increasing noise exposure levels [86,87].

## 5. Conclusions

Our study highlights the potential of α- and β-blockers to counteract some adverse effects of aircraft noise exposure, specifically by improving (micro)vascular function and reducing oxidative stress and inflammation. Although these pharmacological interventions did not alleviate noise-induced hypertension, they effectively reduced vascular oxidative stress markers and mitigated systemic and cardiac inflammation. Improved endothelial function of conductance and microvessels may have implications for vulnerable groups, e.g., patients with pre-established ischemic heart disease or prone to stroke and being under standard α- and β-blocker therapy, since traffic noise was shown to increase the risk of ischemic heart disease [88] and stroke [86,87], and even causing additive risk for recurrent events in patients with coronary artery disease [89]. Additionally, brain studies support the link between noise and heightened oxidative stress and neuroinflammation as a potential link to noise-induced dementia [82,83], reinforcing noise as a potentially significant cardiovascular risk factor.

## Data Availability

Data are all contained within this article. Raw data are available from the corresponding authors upon reasonable request.
